# The Triterpenoid MOMORDIN-Ic Inhibits HCMV by Preventing the Initiation of Gene Expression in Eukaryotic Cells

**DOI:** 10.3390/pathogens13070546

**Published:** 2024-06-28

**Authors:** Eleanor Bradley, Emma Poole, Matthew B. Reeves

**Affiliations:** 1 Institute of Immunity & Transplantation, Division of Infection & Immunity, UCL, Royal Free Campus, London NW3 2PP, UK; eleanor.bradley.20@alumni.ucl.ac.uk; 2 Division of Virology, Department of Pathology, University of Cambridge, Addenbrooke’s Campus, Cambridge CB2 0QQ, UK; elp27@cam.ac.uk

**Keywords:** cytomegalovirus, latency, anti-virals, transcriptional regulation

## Abstract

Human cytomegalovirus (HCMV) primary infection, re-infection, and reactivation from latency cause morbidity in immune-compromised patients. Consequently, potential therapeutic strategies remain of interest for the treatment of infection. Naturally occurring triterpenoids derived from plants have been demonstrated to have anti-viral activity, although their precise mechanisms of action are not always fully understood. Here, we investigate the activity of Mormordin Ic (Mc) and demonstrate that it is potently anti-viral against HCMV. Through investigation of the mechanistic basis of this anti-viral activity, we identify that it is inhibitory to both viral and host gene expression, and to highly induced genes in particular. We go on to observe that Mc impacts on RNA Pol II activity and, specifically, reduces the occupancy of elongating RNA Pol II at a viral promoter. Next, we demonstrate that Mc is inhibitory to HCMV reactivation, and in doing so identify that it has greater activity against the canonical major immediate early promoter compared to the alternative ip2 promoter located downstream. Finally, we see evidence of RNA Pol II occupancy at the ip2 promoter in undifferentiated myeloid cells. Thus, Mc is potently anti-viral and a potential tool to probe the activity of multiple promoters considered important for controlling HCMV reactivation.

## 1. Introduction

Human cytomegalovirus (HCMV) is a major cause of disease in a number of clinical settings [1]. Primary infections, in addition to the reactivation of lifelong latent infections, are sources of morbidity in transplant recipients and congenitally infected infants [1]. Indeed, the health burden associated with HCMV has led it to be designated as highest priority for the development of a vaccine [2]. In lieu of an effective vaccine, the elucidation of additional targets for pharmacological intervention remains an additional priority.

Momordin-Ic (Mc) is one of a family of triterpenoids based on oleanolic acid referred to collectively as saponins [3]. Saponins, and their associated derivatives, are naturally occurring molecules identified in plants that have been demonstrated to possess anti-viral activity against a number of enveloped viruses, but the exact mechanism of action has never been elucidated [4,5]. Indeed, in studies of the related herpes simplex virus (HSV), a spectrum of effects using different saponin derivatives have been reported, including impact on entry, replication, and viral egress at non-toxic concentrations, which suggests that they could have a diverse impact on the viral replication cycle [6].

RNA Pol II regulates eukaryotic gene expression through a complex series of interactions [7]. RNA Pol II is one of three multi-subunit enzymes (RNA Pol I–III) that are responsible for the expression of specific gene subsets within the nucleus [8,9]. The recruitment of RNA Pol II to a promoter, including the HCMV major immediate early promoter (MIEP), typically requires the binding of general transcription factors (GTFs, e.g., TFIID or TATA-binding protein) to the TATA box, and results in the formation of the pre-initiation complex [7]. This complex is then activated by the recruitment of additional GTFs, which are activated in an ATP-dependent manner, ultimately promoting the opening of the DNA helix to allow transcription to initiate. Once initiated, the RNA Pol II complex pauses and requires stabilisation to prevent abortive transcription, with the phosphorylation of key serine residues located within the C-terminal domain (CTD) of RNA Pol II being a critical step in the switch from initiation to efficient elongation [10,11]. Consequently, the successful completion of transcription by RNA Pol II requires many steps that are potentially sensitive to inhibition.

Pertinent to these studies, herpes virus gene expression is dependent on the activity of host RNA Pol II for both lytic infection and latent/reactivation-associated gene expression, although viral encoded factors contribute to RNA Pol II activity particularly at late promoters as well [12,13,14,15,16]. A key promoter that is pivotal for both lytic infection and the transition between latency and reactivation is the MIEP. The MIEP is regulated by chromatin [17,18], with the specific composition dictating the access of specific transcription factors, GTFs, and RNA Pol II, whereby extensive histone acetylation at the MIEP is crucial for HCMV reactivation in dendritic cells (DCs) [19,20]. However, the recent identification of an alternate promoter (intronic promoter 2 (ip2)) located within the MIE intron downstream of the MIEP [21] has revealed that additional complexity in this locus may be important for stimuli and/or cell-type-specific reactivation events when the canonical MIEP is repressed [22,23,24,25].

Here, we show that Mc is a potent inhibitor of HCMV in multiple cell types that support lytic infection, and this is linked to an impact on RNA Pol II activity. We first demonstrate that the inhibitory activity is post-entry and is due to a failure to initiate viral immediate early (IE) gene expression. Cellular fractionation studies show that viral DNA can be detected in the nucleus in the presence of Mc, suggesting that the defect is post-nuclear import. Time-of-addition studies reveal that inhibition is not specific for IE gene expression but, instead, reveals that Mc is a broad-acting inhibitor of the initiation or induction of both viral and eukaryotic gene expression. Interestingly, we see an accumulation of total RNA Pol II at the MIEP in lytic infection in the presence of Mc, but a reduction in RNA Pol II phosphorylated at serine 5. We go on to show that Mc inhibits both the establishment of latency and the induction of IE gene expression in monocyte-derived DCs (MoDCs), and also, but to a lesser extent, PMA-stimulated THP1 cells. Finally, we observe that the inhibitory effect on IE gene expression in PMA-stimulated THP1 cells is disproportionately directed against MIEP but not ip2-derived IE transcripts and, consistent with the proposed mechanism of action of Mc, observe that RNA Pol II is bound to the ip2 promoter in infected THP1 cells prior to PMA stimulation. Taken together, these data demonstrate that Mc is a potent inhibitor of transcription initiation, potentially through reduced RNA Pol II transition to the transcript-elongating form, and could prove a useful pharmacological tool to dissect the differential activity of multiple viral promoters encoded in the MIE region and their individual contributions to HCMV IE gene expression during the initial stages of HCMV reactivation.

## 2. Materials and Methods

### 2.1. Cell and Virus Propagation

Human foetal foreskin fibroblasts (HFFs, SCRC-1041) and adult retinal pigment epithelial 19 cells (ARPE-19, CRL-2302) were purchased from ATCC. All cells were grown in high-glucose Dulbecco’s modified Eagle medium (DMEM, Gibco/Thermofisher, Horsham, UK) supplemented with 10% foetal bovine serum (ThermoFisher, Horsham, UK), 100 U/mL penicillin, and 100 µg/mL streptomycin. For specific experiments, HFFs/ARPE-19 cells were seeded at 80% density 24 h pre-infection on 96- or 24-well coated plates (Corning, Loughborough, UK).

The myelomonocytic cell lines, THP1 (TIB 202; ATCC), were maintained in RPMI-1640 media (Gibco) supplemented with 10% foetal bovine serum (ThermoFisher, Horsham, UK), 100 U/mL penicillin, and 100 µg/mL streptomycin. THP1 cells were maintained at a density of 10^6^/mL to maintain an undifferentiated state prior to use in specific experiments.

To assess viability, HFFs were incubated with Mc at a range of concentrations. Twenty-four hours later, cells were analysed using a CytoTox 96^®^ non-radioactive cytotoxicity assay (Promega, Southampton, UK) to calculate % cytotoxicity, following the manufacturer’s instructions. Toxicity was compared to the DMSO solvent control for each dilution of Mc.

For the viruses and inhibitors, the HCMV isolate TB40/e (a kind gift from Christian Sinzger) was purified from infected human adult retinal pigment epithelial-19 (ARPE-19) cells and then amplified for one round in human fibroblasts. Supernatants from HFF cultures were purified using sorbitol gradient (20% D-sorbitol, 50 mM pH7.4 Tris, 1 mM MgCl_2_) and centrifuged (65,000× *g* for 90 min at 4 °C) using an SW32Ti rotor (Beckman Coulter, High Wycombe, UK) in an Optima XE ultracentrifuge (Beckman Coulter, High Wycombe, UK). Purified virus was characterised by TCID50 on the ARPE-19 cells and HFFs.

Momordin Ic (Stratech Scientific, Ely, UK) was dissolved in DMSO and used at 2 μM in all experiments following titration to find the optimal dose that inhibited infection. 4,4′-Diisothiocyano-2,2′-stilbenedisulfonic acid (DIDS; Sigma-Aldrich, Darmstadt, Germany) was dissolved in DMSO and used as previously described at 10 μM [26]. Heparin was dissolved in PBS and used at 10 ug/mL.

### 2.2. Latency and Reactivation Experiments

CD14+ monocytes were isolated from venous blood donations from healthy volunteers using Ficoll separation and MACS CD14+ positive cell separation (Miltenyi Biotec, Surrey, UK) before being seeded on plastic and subsequently fed with X-VIVO-15 supplemented with 2 mM L-glutamine. After 24 h, cells were infected with HCMV TB40/e at an MOI equivalent to 5 on HFFs (routinely, about MOI 0.5 on ARPE-19 cells). At 3 days post-infection, cells were differentiated with GM-CSF/IL-4 (both 1000 U/mL; Peprotech/ThermoFisher, Horsham, UK) for 6 days and a further 3–24 h with IL-6 (500 ng/mL; Peprotech/ThermoFisher, Horsham, UK) to promote reactivation.

Alternatively, THP1 cells were infected with HCMV TB40/e at an MOI equivalent to 5 on HFFs (routinely, about MOI 0.5 on ARPE-19 cells) and then cultured in RPMI-1640 supplemented with FCS (2%) to limit cell replication. At 5 days post-infection, cells were activated with PMA (20 nM; SIGMA) for 3–48 h to promote viral reactivation.

### 2.3. Nucleic Acid Isolation and Analysis

DNA was extracted by proteinase K digestion. Cells were treated with 100 mM KCl, 10 mM Tris-HCl, and 2.5 mM MgCl for 5 min prior to the addition of an equal volume of 10 mM Tris-HCl, 2.5 mM MgCl, 1% *v*/*v* Tween-20, 1% *v*/*v* Nonidet P-40 (Santa Cruz Biotechnology, Santa Cruz, USA), and 0.4 mg/mL Proteinase K (Sigma-Aldrich, Darmstadt, Germany) for a further 5 min. The resultant mixture was heated for 1 h at 60 °C followed by 10 min at 95 °C. DNA was amplified using primers specific for viral (UL138 F: 5′ GAG CTG TAC GGG GAG TAC GA, R: 5′ AGC TGC ACT GGG AAG ACA CT), nuclear (F: 5′ AGG GTA TCT GGG CTC TGG and R: 5′ GGC TGA AAA GCT CCC GAT TAT), or mitochondrial (F: 5′ ACA CCC TCC TAG CCT TAC TAC and R: 5′ GAT ATA GGG TCG AAG CCG C) DNA.

To isolate RNA, cells were washed once with PBS before the direct harvest of the samples with RLT buffer (Qiagen, Manchester, UK). RNA extraction was then performed as per the manufacturer’s instructions using the RNeasy Mini Kit (Qiagen, Manchester, UK), and then converted to cDNA using a Quantitect reverse transcription kit (Qiagen, Manchester, UK) as described by the manufacturer. DNA or cDNA was then amplified by Sybr green (Thermofisher, Horsham, UK) qPCR using previously published gene-specific primers: UL123 5′-GCG CCA GTG AAT TTC TCT TC and 5′-ACG AGA ACC CCG AGA AAG ATG 3′; MIEP-derived IE 5′-TTG ACC TCC ATA GAA GAC AC 3′ and 5′-AGG ACT CCA TCG TGT CAA GG 3′; ip2-derived (UTR70) 5′-TAG CTG ACA GAC TAA CAG AC 3′ and 5′-AGG ACT CCA TCG TGT CAA GG-3′; 18S 5′-GTA ACC CGT TGA ACC CCA 3′ and 5′-CCA TCC AAT CGG TAG CG-3′; GAPDH, 5′-GGA AGC TTG TCA TCA ATG and 5′-CCC CAC TTG ATT TTG GAG; Actin 5′-GCT CCG GCA TGT GCA and 5′-AGG ATC TTC ATG AGG TAG T; UL44 5′-GTA CAA CAG CGT GTC GTG CT-3′ and 5′-ATA ACC GCG TCA GTT TCC AC-3′; UL138 (F: GAG CTG TAC GGG GAG TAC GA, R: AGC TGC ACT GGG AAG ACA CT), IFIT2 (F: ACT GCT GAA AGG GAG CTG AA, R: TGC ACA TTG TGG CTT TGA AT), IFIT3: (F: AGA AAT GAA AGG GCG AAG GT, R: ATG GCC TGC TTC AAA ACA TC), CXCL10: (F: TGG CAT TCA AGG AGT ACC TC, R: TTG TAG CAA TGA TCT CAA CAC G).

Where possible, relative expression was analysed using the 2delta delta Ct method, comparing the control with the test sample. Alternatively, to express absolute values in the qPCR analyses, 2delta Ct was used to represent signals above background signals in the qPCR.

### 2.4. Chromatin IP and RNA Pol II Binding

Cells were fixed with 1% PFA for 10 min then washed in glycine buffer. Chromatin was then prepared using a ChIP-IT express kit (Active motif/Cambridge Bioscience, Cambridge, UK). Antibodies were directed against the CTD of RNA Pol II (ab26721; abcam, Cambridge, UK) or serine 5 phosphorylated from RNA Pol II (ab5131; abcam, Cambridge, UK), or the rabbit isotype control with 5 ug of antibodies on chromatin prepared from 10^6^ cells.

### 2.5. Indirect Immune-Fluorescent Staining for Viral Gene Expression

Cells were fixed by treatment with 100% ice-cold ethanol for >20 min at −20 °C. To visualise infection, cells were then washed in PBS and incubated with mouse anti-IE (clone 6F8.2, Merck Millipore, Darmstadt, Germany; 1:2000 dilution) for 1 h, followed by incubation with Goat anti-Mouse IgG-Alexa-fluor-594 nm (Life Technologies/Thermofisher, Horsham, UK; 1:2000 dilution) plus 0.5 µg/mL DAPI for 1 h. Infection was quantified using Hermes WiScan technology (IDEA-Bio, Rehovo, Israel) and quantified using Metamorph software Molecular Devices, Berkshire, UK).

### 2.6. Statistical Analyses

Either a one-way ANOVA (analysis of variance) or Kruskal–Wallis test was applied to test for variance within the means, and then specific post hoc tests were performed to identify specific means with statistical significant differences. For the comparison of two samples, a Mann–Whitney test or the standard t test with Welch correction was applied. Statistical analyses were only applied if *n* > 2. All bar charts depict the mean and one standard deviation from the mean. Significance was assumed if *p* < 0.05.

## 3. Results

### 3.1. Pre-Treatment of HFFs with Mc Blocks HCMV Lytic IE Gene Expression at Post-Entry Stage

**Figure 1:**
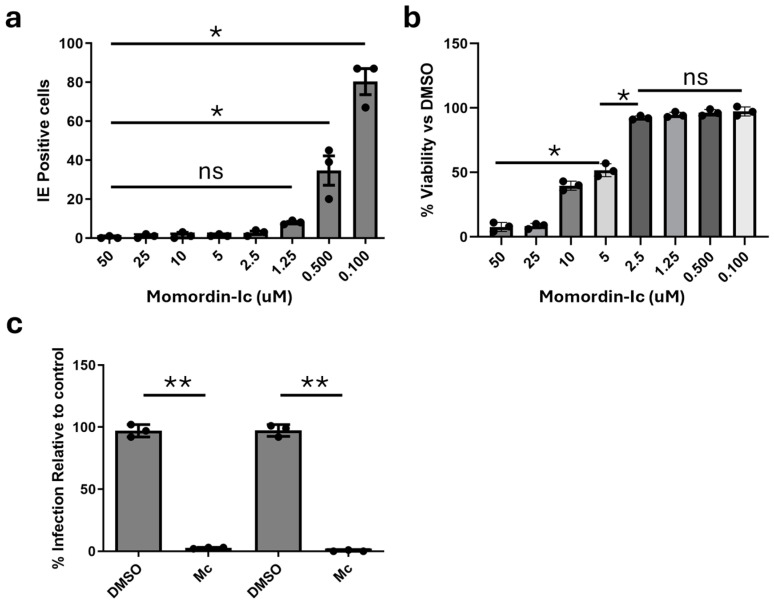
Momordin-Ic inhibits viral infection of permissive cells in a single round infection. (**a**,**b**) HFFs were pre-treated with serial dilutions of Mc (50 μM–100 nM) and then infected in presence of drugs and stained for IE protein expression 24 hpi (**a**) or assayed for cell viability 24 h later (**b**). Statistical analysis was performed using Kruskal-Wallis with Dunn’s multiple comparison test. * *p* < 0.05; ns non-significant. (**c**) ARPE and THP1-macrophage cells were pre-treated with Mc (2 μM), infected in presence of drugs, and then stained for IE protein expression 24 hpi. Infection was quantified by Hermes WiScan automated analysis. Statistical analysis is unpaired *t* test with Welch’s correction. ** *p* < 0.01.

To investigate whether Mc had any anti-viral activity, we began our studies on lytic infection. HFFs were pre-incubated with Mc over a dose range and infected with HCMV at MOI = 5. Then, infection was measured 24 h later by IF. The data show that Mc potently inhibited HCMV infection in HFFs, with similar inhibitory activity being observed at 1.25 μM (Figure 1a). Furthermore, when used at a concentration inhibitory but not toxic in HFFs (Figure 1b), it was also a potent inhibitor of ARPE-19 infection and THP1 macrophages (Figure 1c).

**Figure 2:**
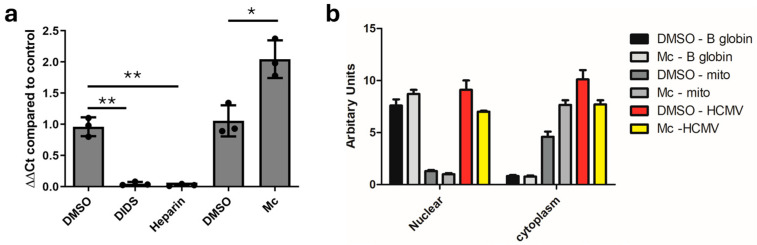
Momordin Ic works post-viral entry. (**a**) HFFs were pre-treated with DMSO (solvent control), DIDS, Heparin, DMSO (solvent control) or 2 μM Momordin Ic (Mc) and DNA was isolated 3 hpi and amplified by qPCR and expressed relative to untreated control. Statistical analysis by ANOVA followed by Tukey’s post multiple comparisons test was performed. * *p* < 0.05; ** *p* < 0.01. (**b**) HFFs were pre-treated with DMSO (control) or 2 μM Momordin Ic (Mc) and cells were fractionated into nuclear and cytoplasmic fractions. DNA was isolated and analysed for viral (HCMV), nuclear (B globin) and mitochondrial (mito) DNA. Values were calculated as difference of Ct from background PCR signal (arbitrary units).

One potential explanation was that the delivery of viral genomes to cells pre-treated with Mc was impaired. To test this, HFFs pre-treated with Mc were infected with HCMV and then after 3 h washed in PBS and analysed by qPCR. The data clearly show that intracellular DNA genomes were present at similar (and potentially slightly higher) levels irrespective of Mc pre-treatment, in contrast to known viral binding and entry inhibitor heparin or 4,4′-diisothiocyano-2,2′-stilbenedisulfonic acid (DIDS) (Figure 2a), which we have previously shown to block HCMV entry [26]. Furthermore, fractionation experiments confirmed that viral DNA was detectable in the nuclei in infected cells irrespective of whether they were pre-treated with Mc or not (Figure 2b), suggesting that inhibitory activity was being exerted post-nuclear entry but pre-IE protein expression.

### 3.2. Momordin Ic Inhibits Viral Gene Transcription When Added Post-Entry

**Figure 3:**
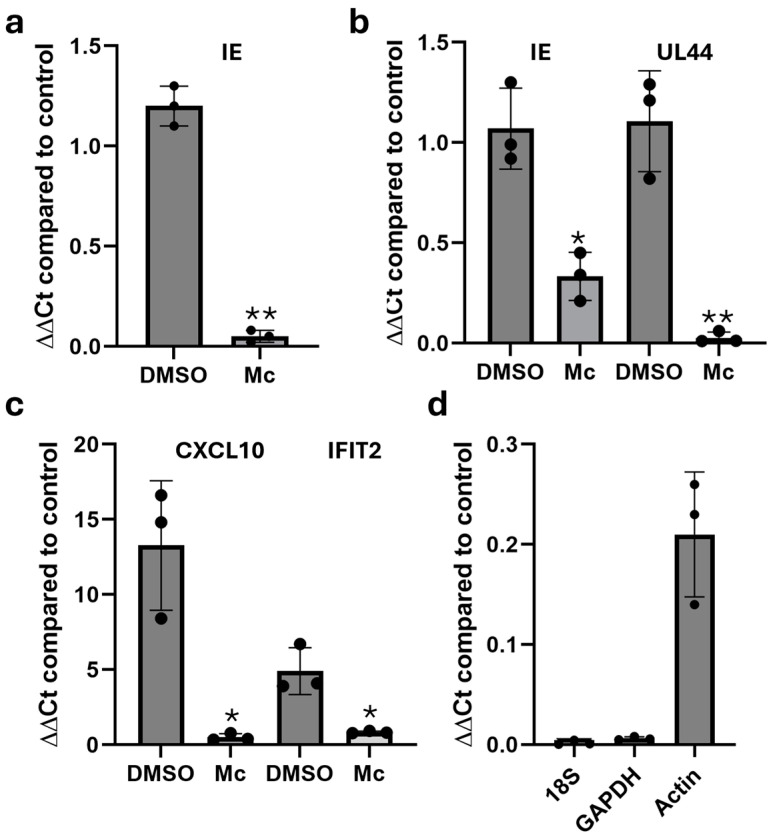
Momordin-Ic inhibits viral and eukaryotic transcription. (**a**) HFFs untreated (control) or pre-treated with DMSO or 2 μM Momordin Ic (Mc) were analysed for IE RNA expression by qPCR. (**b**) HFFs were infected with HCMV and then incubated with DMSO or 2 μM Momordin Ic (Mc) 8 hpi. IE RNA expression was then analysed 24 hpi by qPCR. (**c**) THP1 cells were either untreated (control) or pre-treated with DMSO or 2 μM Momordin Ic (Mc) and then stimulated with IFNb (1000 U/mL). After 8 h, RNA was analysed for CXCL10 and IFIT2 expression by qPCR and expressed relative to expression in untreated, unstimulated control cells. (**d**) HFFs were incubated with DMSO (control) or 2 μM Momordin Ic and then analysed for 18S, GAPDH and actin expression by qPCR after 24 h. Expression in Momordin Ic treated cells relative to DMSO is shown. *n* = 3 for all experiments. Statistical analysis (**a**–**c**) was performed using an unpaired t test with Welch correction. * *p* < 0.05; ** *p* < 0.01.

Next, we investigated the impact of Mc on transcription using time-of-addition studies. Consistent with the protein IF data, pre-treatment with Mc dramatically inhibited IE RNA expression (Figure 3a). In contrast, if the inhibitor was added 6 hpi, we observed that the impact on IE transcript levels was markedly reduced when analysed 24 hpi. Notably though, there was a reduction in UL44 transcript levels under these conditions (Figure 3b).

Taken together, these data clearly suggest that Momordin Ic is anti-viral due to a potent blockade of viral RNA expression, with the time of addition dictating which viral transcriptional class the effect is most overt in. However, although the data point to a clear inhibition of viral transcription, it was evident throughout our analyses that actin mRNA levels were also routinely lower in the Mc-treated samples if equivalent amounts of RNA were analysed. Thus, this also potentially suggests that we were underestimating the impact on viral transcription since we were using the 2ΔΔCT method of analysis. Subsequently, we investigated how virus-specific these effects were, and thus decided to investigate the induction and expression of host gene expression in more detail. The data show that Mc also blocked interferon-stimulated gene (ISG) expression in response to IFNb (Figure 3c). Furthermore, Mc had a dramatic impact on 18S expression, and clear but smaller effects on GAPDH and actin (Figure 3d). These data suggest the effects were most pronounced at loci where transcription was being induced or highly active (e.g., de novo transcription from viral promoters, IFN-induced genes, and even RNA Pol I-dependent rRNA expression (e.g., 18S), which accounts for ~60% of all transcripts in eukaryotic cells [27]).

### 3.3. Reduced Binding of Phosphorylated RNA Pol II to the MIEP in the Presence of Momordin Ic

**Figure 4:**
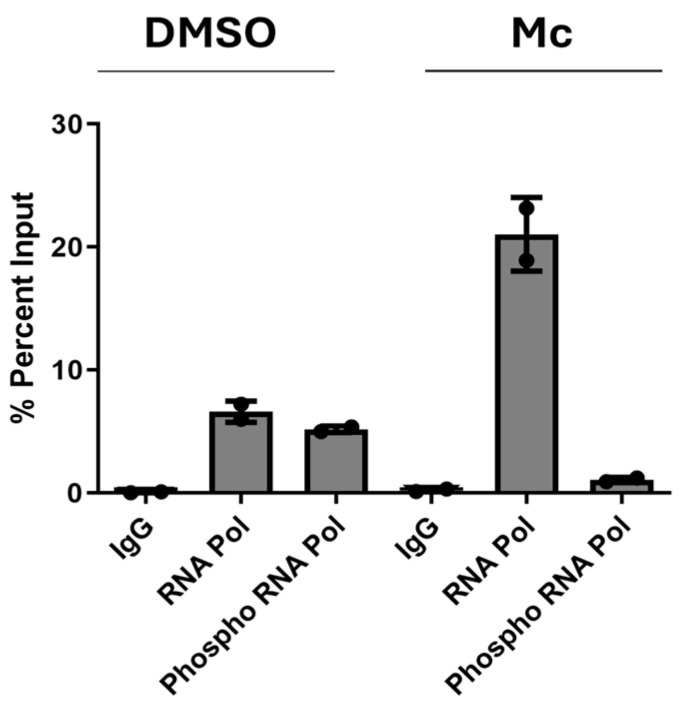
Momordin Ic reduces occupancy of phosphor RNA Pol II at the MIEP. HFFs were pre-treated with DMSO or 2 μM Momordin Ic (Mc), infected and then subjected to ChIP analysis 3 hpi with antibodies against RNA Pol II, phosphorylated RNA Pol II or an isotype-matched control. Samples were analysed by qPCR with MIEP primers and values expressed as %Input. *n* = 2.

To understand the molecular basis for the inhibition of transcription by Mc, we focused on the regulation of the MIEP. We hypothesised that the inhibition of transcription could be mediated via a block to RNA Pol II recruitment to the MIEP. To investigate this, infected HFFs which had been pre-treated for 3 h with Mc were then analysed for RNA Pol II binding to the MIEP by ChIP at 3 hpi. The data show that RNA Pol II occupancy at the MIEP was actually higher in the presence of Mc and not reduced (Figure 4). However, a concomitant analysis of RNA Pol II phosphorylated at the CTD on serine 5 clearly showed a decrease in occupancy at the MIEP in Mc-treated cells (Figure 4).

### 3.4. Momordin Ic Inhibits MIE Promoters in a Time- and Cell-Type-Dependent Manner during Reactivation

The capacity of Mc to inhibit the induction of MIE gene expression during lytic infection led us to investigate it as an inhibitor of MIE gene expression during the early stages of HCMV reactivation.

**Figure 5:**
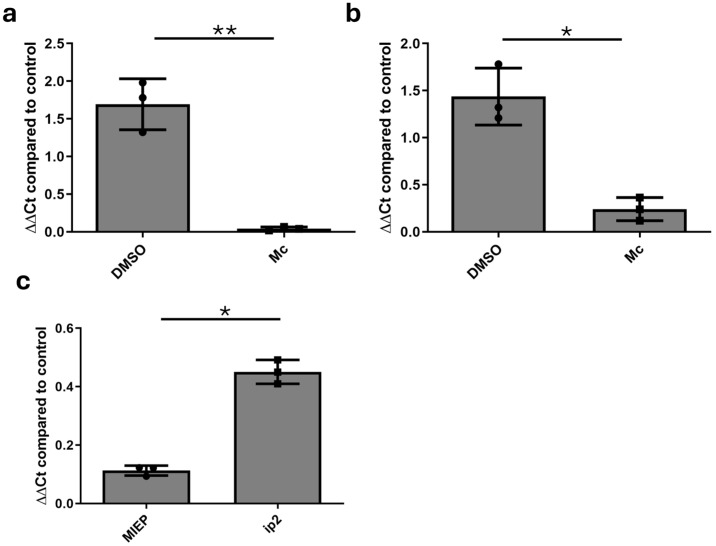
Momordin Ic inhibits IE expression in reactivating cells. (**a**,**b**) Latently infected monocyte-derived DCs (**a**) or THP1 cells (**b**) were either untreated (control), or pre-incubated with DMSO or 2 μM Momordin Ic (Mc) for 3 h prior to IL-6 (**a**) or PMA (**b**) stimulation. After 24 h, cells were analysed for IE gene expression relative to untreated control. (**c**) RNA harvested in (**b**) was subsequently analysed for MIEP and ip2-derived IE transcripts by qPCR and Mc samples expressed relative to DMSO solvent control. Statistical analysis was performed using a Mann-Whitney comparison of independent pairs. * *p* < 0.05; ** *p* < 0.01. *n* = 3.

To carry this out, we generated latently infected monocytes and differentiated them to immature monocyte-derived dendritic cells (MoDCs). The incubation of these cells with IL-6 will then promote HCMV reactivation [28], with the first event being the induction of IE gene expression. Thus, we took immature MoDCs and, prior to IL-6 stimulation, incubated them with Mc for 3 h. Six hours post-IL-6 stimulation, we isolated RNA from the cells and analysed them for IE RNA expression by qPCR. The data clearly show that Mc potently inhibited HCMV IE gene expression induction via IL-6 (Figure 5a). Next, we analysed the impact of Mc in the THP1 macrophage model. Here, the reactivation of latently infected THP1 cells can be triggered by stimulation with the phorbol ester, PMA. Thus, prior to PMA stimulation, infected THP1 cells were incubated with Mc for 3 h. As observed in MoDCs, IE transcription was reduced in the presence of Mc (Figure 5b), but not as overtly as observed in MoDCs (Figure 5a). Next, we performed a more detailed analysis of the origin of the IE transcripts, with both the canonical MIEP and the alternate ip2 promoters having been demonstrated as potential sources of IE transcripts. The analysis of IE transcription in PMA-stimulated THP1 cells using a differential qPCR for specific transcripts revealed that the impact of Momordin Ic was disproportionately directed against the MIEP and not the ip2-promoter-derived transcripts, although a substantial reduction in both was still observed (Figure 5c).

### 3.5. RNA Pol II Is Bound to ip2 Promoter in Latently Infected THP1 Cells

**Figure 6:**
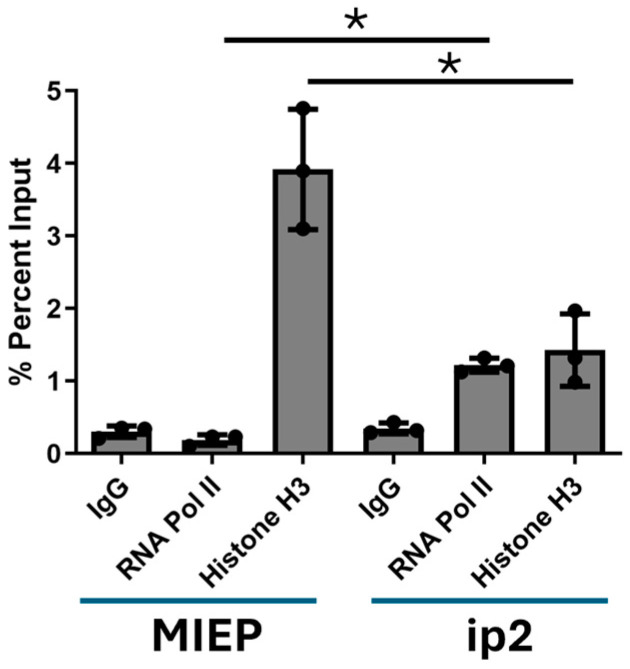
RNA Pol II binding to ip2 promoter region in latently infected cells. THP1 cells were infected with HCMV and after 5 days subjected to ChIP analysis with antibodies against RNA Pol II, histone H3 or a rabbit isotype-matched control. DNA was then analysed by qPCR with primers directed against MIEP and intron A (ip2) sequences. Statistical analysis was performed using a Mann-Whitney comparison of independent pairs. * *p* < 0.05. *n* = 3.

Finally, we investigated the occupancy of RNA Pol II on the MIE region in latently infected THP1 cells via a ChIP assay (Figure 6). The data show that a basal level of RNA Pol II binding to ip2 could be detected in these cells prior to stimulation with PMA but, in contrast, RNA Pol II occupancy was rarely detected at the MIEP (Figure 6), suggesting that the impact on transcription from the MIEP was more pronounced due to some ip2 promoters being potentially pre-loaded with RNA Pol II. Finally, it was interesting to observe that histone H3, which served as a positive control, appeared enriched on the MIEP versus the ip2 promoter region (Figure 6).

## 4. Discussion

In the current study, we have investigated the anti-viral activity of the triterpenoid Mc against HCMV and demonstrated that it potently blocks lytic infection and reactivation at multiple stages of the virus lifecycle. The anti-viral activity is likely linked to a general inhibition of transcription and, specifically, manifests as a reduction in the association of active RNA Pol II with the viral MIEP. Notably, these effects were not MIEP-specific; instead, Mc appeared to potently block the induction of multiple viral RNAs depending on the time of addition. The analogous impact on inducible cellular RNA expression also likely explains the general toxicity associated with the compound when added to cells for a prolonged period of time (i.e., 36 h onwards), which is likely due to the general impact on host transcription.

The recruitment and subsequent activation of RNA Pol II at promoters is complex. Our ChIP data suggest that RNA Pol II recruitment was not necessarily impaired but, instead, demonstrated a failure to transition to the active form following proximal promoter pausing. Triterpenoids (of which momordin Ic is a member) are just one family of a range of compounds that can inhibit transcription [29]. Indeed, the unrelated Actinomycin D has been used extensively as a transcriptional inhibitor, including in the original annotation of the temporal cascade of herpes virus gene expression [30]. Actinomycin D is proposed to bind competitively to DNA, and thus prevent RNA Pol II binding and elongation [31]; therefore, it is possible Mc could work in a similar way. However, distant triterpenoids have also been shown to inhibit DNA polymerase and DNA topoisomerase II activity, but via binding to the polymerase directly in the DNA binding domain [32]. However, the data presented suggest that the recognition of DNA is not impaired by RNA Pol II—the defect appeared to be in the transition to the elongating form, although we cannot rule out that Mc destabilises the elongating form of RNA Pol II.

Interestingly, another reported target of Mc is SENP1 (sentrin protease 1), a nuclear localised member of a family of related sentrin proteases that has been suggested as the basis of the anti-cancer activity of Mc [33,34]. Pertinently, SUMO inhibits both RNA Pol I and RNA Pol II transcription through the prevention of elongation [35,36]. Thus, it is possible that the inhibition of the specific SENP1 activity (one of six SENPs in the cell that have deSUMOylase activity [37,38]) could explain this phenotype of Mc, although this is tempered by the observation that Mc activity against SENP1 is reported at higher concentrations [33]. Furthermore, HCMV itself encodes evolutionarily distinct deSUMOylase (LUNA) during latency, which could also be an important target [39]. Future studies could investigate whether Mc at the concentration used in these studies has any activity against LUNA. This would be interesting for determining how specific Mc is for SENP1 versus other classes of deSUMOylases (both viral and cellular).

The toxicity of Mc, if used for prolonged periods of time, precludes studies of it’s potential as an anti-viral,, the observation that Mc reduced RNA Pol II occupancy on promoters provided the opportunity to investigate the complex mechanisms controlling the reactivation of HCMV IE gene expression. We and others have observed that MIE gene expression can be induced following the activation of the canonical MIEP or alternate internal promoters within the MIE region (e.g., ip2) in a cell-type- and ligand-specific fashion [22,23,24,25]. A prior study suggests a model whereby MIEP-derived IE transcription from latency requires substantial chromatin remodelling to initiate gene expression [40]. In contrast, we hypothesise that the activation of the ip2 promoter is often seen when MIEP activity is impaired [24]. Here, we report that, in DCs stimulated with IL-6 to promote IE gene expression (which we argue is predominantly MIEP-dependent), we observe that Mc potently blocks IE gene expression. Similarly, in THP1 cells, MIEP-derived transcription was impaired, particularly in contrast to that observed with ip2-derived transcripts.

One potential interpretation, based on the data we present here within the context of our understanding of RNA Pol II activity, is that at least some of the ip2 promoters are poised with RNA Pol II loaded, or represent promoters with basal activity which again have sufficient RNA Pol II occupancy/activity to initiate gene expression, even in the presence of Mc. Indeed, RNA Pol II occupancy has previously been reported in the gene regions of the MIE locus during latency, which could be consistent with this hypothesis [41]. Countering this idea are the data that argue that the biggest impact of Mc is seen on the relative levels of phosphorylated RNA Pol II. This suggests that the transition from proximal pausing to elongation is the target, and thus why that would have a greater impact on the MIEP than ip2 is unclear. One explanation may be the non-canonical nature of histone deposition on HCMV genomes in both lytic and latent infection, which may suggest subtle differences in how RNA Pol II interacts with viral genomes versus eukaryotic genomes [13,42]. For example, it has long been understood that chromatin is inhibitory to the transition of RNA Pol II to its active form after promoter pausing [43,44]. The observation that less histone H3 was associated with intron A versus the MIEP coupled with RNA Pol II occupancy at ip2 prior to reactivation may suggest some ip2 promoters have lower thresholds for reactivation overall, and why the impact of Mc was greater against the MIEP. Further studies using phosphor-specific antibodies against multiple RNA Pol II serine residues in the CTD may help to understand this further.

In summary, we have reported the characterisation of an inhibitor of HCMV infection that reduces elongated RNA Pol II occupancy on induced promoters, resulting in a substantial inhibition of viral gene expression. Interestingly, the use of this compound to probe transcription during the early stages of viral reactivation revealed that it disproportionately inhibited induced gene expression from the MIEP compared to the ip2 promoter, which we hypothesise is potentially due differences in RNA Pol II association with the MIEP and ip2 during viral latency and reactivation.

## Data Availability

No large datasets were generated in this study. Specific datasets are available from the corresponding author on request.
